# Caffeine Modulates Food Intake Depending on the Context That Gives Access to Food: Comparison With Dopamine Depletion

**DOI:** 10.3389/fpsyt.2018.00411

**Published:** 2018-09-06

**Authors:** Mercè Correa, Noemí SanMiguel, Laura López-Cruz, Carla Carratalá-Ros, Régulo Olivares-García, John D. Salamone

**Affiliations:** ^1^Àrea de Psicobiologia, Campus de Riu Sec, Universitat Jaume I, Castelló, Spain; ^2^Behavioral Neuroscience Division, University of Connecticut, Storrs, CT, United States

**Keywords:** anxiety, appetite, methylxanthine, decision-making, sucrose, tetrabenazine

## Abstract

Caffeine is a methylxanthine consumed in different contexts to potentiate alertness and reduce fatigue. However, caffeine can induce anxiety at high doses. Caffeine is also a minor psychostimulant that seems to act as an appetite suppressant, but there are also reports indicating that it could stimulate appetite. Dopamine also is involved in food motivation and in behavioral activation. In the present series of experiments, we evaluated the effects of acute administration of caffeine on food consumption under different access conditions. CD1 male adult mice had access to highly palatable food (50% sucrose) in a restricted but habitual context, under continuous or intermittent access as well as under anxiogenic, or effortful conditions. Caffeine (2.5–20.0 mg/kg) increased intake at the highest dose under familiar continuous and intermittent access. However, this high dose reduced food intake in the dark-light paradigm. In contrast, a dopamine-depleting agent, tetrabenazine (TBZ; 1.0–8.0 mg/kg) did not affect food intake in any of those experimental conditions. In the T-maze-barrier task that evaluates seeking and taking of food under effortful conditions, caffeine (10.0 mg/kg) decreased latency to reach the food, but did not affect selection of the high-food density arm that required more effort, or the total amount of food consumed. In contrast, TBZ (4.0 mg/kg) reduced selection of the high food density arm with the barrier, thus affecting amount of food consumed. Interestingly, a small dose of caffeine (5.0 mg/kg) was able to reverse the anergia-inducing effects produced by TBZ in the T-maze. These results suggest that caffeine can potentiate or suppress food consumption depending on the context. Moreover, caffeine did not change appetite, and did not impair orientation toward food under effortful conditions, but it rather helped to achieve the goal by improving speed and by reversing performance to normal levels when fatigue was induced by dopamine depletion.

## Introduction

Caffeine is the psychostimulant substance most widely consumed in the world, and it is found in several types of food and beverages (1). Psychostimulants are characterized by stimulation of locomotion and feelings of invigoration and increase in perceived energy, although at high doses they can induce stereotypies and anxiety (2–5). This category of drugs is known for its anorectic effects, and in fact caffeine is a common constituent in over-the-counter weight-loss supplements (6). However, caffeine in humans does not seem to have a consistent pattern of effects on appetite and energy intake. While some studies report that it exerts a slight anorectic effect (7), others do not report significant changes (8, 9).

In animal studies, the literature shows also a complex pattern of results. In non-deprived mice, a recent study demonstrated that acute administration of caffeine (6.0–24.0 mg/kg) increases intake of standard free chow, and that animals are not more activated or anxious (10). In different types of operant conditions imposed on food restricted rats, acute doses of caffeine produce dose dependent effects; doses of 40.0 mg/kg or higher reduced lever pressing for different types of food (11, 12), and doses up to 25.0 mg/kg increased lever pressing independently if there was no net increase on food access (12–14).

Nucleus accumbens (Nacb) dopamine (DA) has been implicated in some features of food motivation (15–18). Caffeine does not clearly induce Nacb DA release (19, 20). However, it is a non-selective adenosine receptor antagonist, and these receptors have a functional interaction with DA receptors in striatum. Adenosine receptors are co-localized with DA receptors converging into the same intracellular cascade in an opposite way; while A_1_ receptors are co-localized with D_1_ receptors, A_2A_ receptors are co-localized with D_2_ receptors in different groups of striatal neurons (21–23). Thus, antagonism of adenosine receptors leads to the opposite effect to DA antagonism. Several previous studies have focused on the functional interaction between adenosine and DA receptors in studies of effort-related decision-making processes that allow access to different quantities or types of food in food restricted animals (15, 24–26). For example, in a T-maze procedure in which mice have to jump a barrier to get access to a relatively large quantity of food, co-administration of theophylline, another methylxantine that acts as a non-selective adenosine antagonist, reversed the anergia inducing effects of a DA D_2_ receptor antagonist, restoring normal levels of effort that lead to more food (26).

In the present studies, we wanted to assess the impact of a broad range of doses of caffeine (2.5–20.0 mg/kg) on highly palatable food intake in mice. Because anxiety has been shown to suppress appetite, we compared the impact of caffeine on food intake under habitual conditions vs. anxiogenic conditions. Since caffeine invigorates behavior, we also studied intake of palatable food when effort was a component of the decision-making process, such that food restricted mice could gain access to higher quantities of food by exertion of effort. In addition, we also evaluated the ability of caffeine to reverse the effects of a DA-depleting agent that reduces willingness to work for food (anergia). We also assessed if this DA-depleting agent changes, by itself, food intake under habitual, anxiogenic or effortful conditions. These results could clarify the potential therapeutic effects of caffeine on appetite under normal or pathological conditions.

## Materials and methods

### Subjects

CD1 male mice (24–28 g) purchased from Janvier, France S.A. were 4 weeks old upon arrival to the laboratory (*N* = 47), and after a week of adaptation to the colony conditions, all experiments started. Mice were housed in groups of three animals per cage with tap water and standard chow food available *ad libitum* in the home cage across the entire experiment, except in experiments 5–7, in which mice were food-restricted in their home cage (to a maximum of 85% free feeding body weight) throughout the study. These animals received between 7 and 8 g of standard chow food per cage during the week days and between 13 and 14 g during the weekend days to allow normal growth. The colony was kept at a temperature of 22 ± 2°C with lights on from 08:00 to 20:00 h. All animals were covered by a protocol approved by the Institutional Animal Care and Use committee of Universitat Jaume I. All experimental procedures complied with directive 2010/63/EU of the European Parliament and of the Council, and with the “Guidelines for the Care and Use of Mammals in Neuroscience and Behavioral Research,” National Research Council 2003, USA. All efforts were made to minimize animal suffering, and to reduce the number of animals used.

### Pharmacological agents

Caffeine (1,3,7-trimethylxanthine; Sigma-Aldrich, Spain) was dissolved in 0.9% w/v saline and was administered 30 min before testing. Saline solution was used as its vehicle control. The range of caffeine doses (2.5, 5.0, 10.0, and 20.0 mg/kg) was selected based on previous and pilot studies (27). Tetrabenazine (TBZ) [(*R,R*)-3-Isobutyl-9,10-dimethoxy-1,3,4,6,7,11b-hexahydro-pyrido[2,1-*a*]isoquinolin-2-one] (CIMYT Quimica SL, Spain), administered 120 min before testing, was dissolved in a vehicle solution of 0.9% saline (80%) and dimethylsulfoxide (DMSO; 20%) (pH = 4.5). DMSO was used as vehicle control. All solutions were administered intraperitoneally (IP).

### Apparatus and testing procedures

The same type of food was used in all the experiments; 45 mg (experiments 1–4) or 20 mg (experiments 5–7) precision pellets for rodents (TestDietTM) with a balanced nutrient composition and a 50% sucrose content that gave it palatable characteristics.

#### Palatable food consumption under habitual conditions: continuous or intermittent access

**Figure 1:**
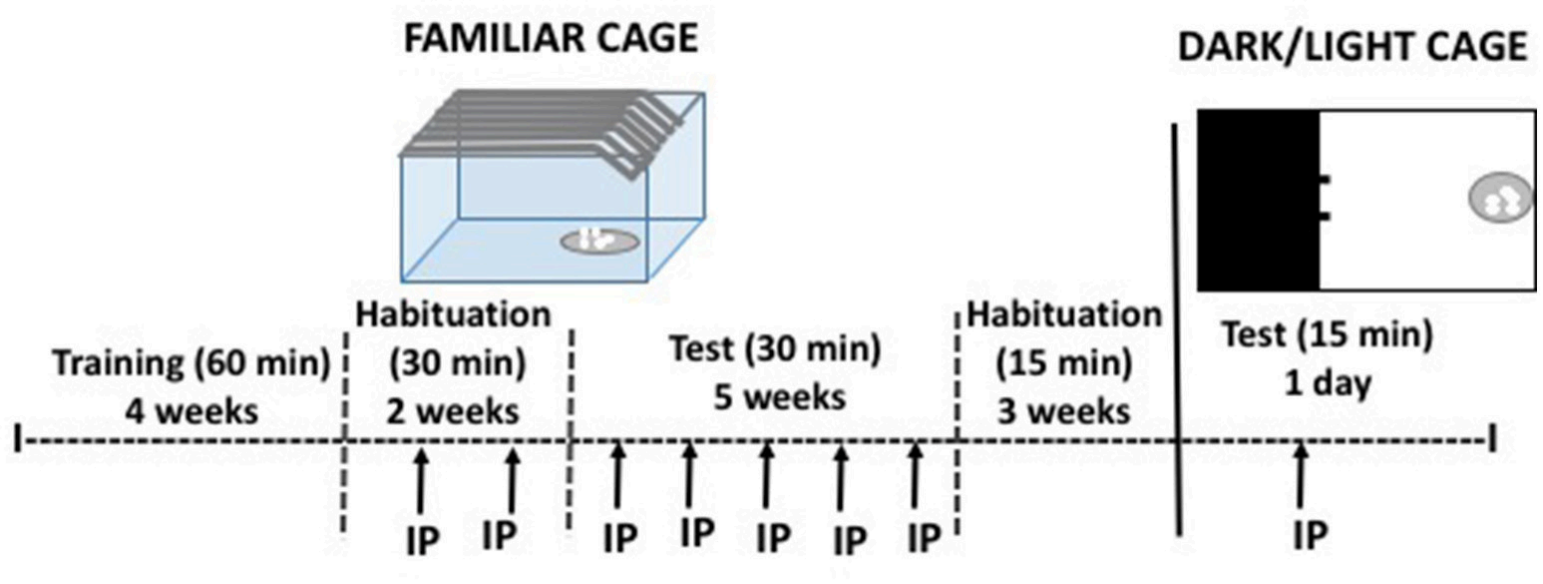
Schematic representation of the cage for habitual and limited food access, and the DL apparatus used in experiments 1–4, with the experimental procedure.

During 4 weeks (5 days per week) non-food restricted mice were placed individually in standard home cages where they had *ad libitum* access to highly palatable pellets (45 mg each). Baseline sessions lasted 60 min (data were registered every 30 min), starting 3 h prior the beginning of the dark cycle. Thus, animals consumed food in a familiar and repetitive condition. During 2 more weeks, animals were habituated to receive an IP vehicle injection once a week, and sessions lasted 30 min (see Figure 1). Because it has been suggested that non-continuous access to food increases food consumption leading to binge eating, we had two conditions of food in a familiar context: the continuous group had sessions 5 days per week (Monday to Friday), and the intermittent access group had sessions 3 days per week (Monday, Wednesday and Friday). The test phase lasted five more weeks during which each subject received all doses of caffeine (Experiments 1 and 2) or TBZ (Experiments 3 and 4) in a randomly varied order, once a week. Body weight was registered 3 times per week.

#### Palatable food consumption under anxiogenic conditions

After completing the habitual food-consumption experiments, the same mice had access to the highly palatable food for three additional weeks of baseline with no treatment, and shorter sessions (15 min) (see Figure 1). Then, mice in the continuous and in the intermittent conditions were divided in two treatment groups: saline or 20 mg/kg of caffeine (Experiment 2), and DMSO or TBZ (8 mg/kg; Experiment 4). Animals that had received caffeine in the previous experiment also received caffeine in the present one, and the same was true for animals that had received TBZ. Doses were selected based on their impact in the previous experiments. In one single test day, animals were placed for 15 min in a dark and light (DL) paradigm. In the DL box, one chamber was enclosed and dark, and the open chamber where the food dish was placed, was intensely illuminated. Tests started when each subject was placed in the dark chamber. Since there were no differences in food intake in the DL between animals in the intermittent and the continuous groups data from both groups were pooled in order to increase the number of animals per group. The amount of food (mg) consumed during 15 min was recorded, as well as the time spent in the lit compartment.

#### Effort-based decision-making for palatable food in a T-Maze with barrier

In the last group of experiments (5–7), we studied the impact of caffeine or TBZ in the T-maze procedure that imposes an effort restriction in order to get access to higher quantities of food. In order to make animals to learn and perform the task, mice were food restricted in their home cage. This procedure is based on previous published work (26). The T-maze apparatus consisted of a central corridor with two opposed arms (see Figure 4). Each arm provided a different density of food: 2 pellets (20 mg each) in the high density (HD) arm, and 1 pellet (20 mg) in the low density (LD) arm. Pellets were located in dishes placed near the far walls of the maze arms. The HD arm contained a vertical barrier (12 cm high) that provided the effort-related challenge. Half the mice had the HD arm with the barrier consistently located on the left side, while half the mice had the HD arm and barrier on the right side. During the first training phase no barrier was present, and for the first 2 days of the initial training, mice had free access to both arms of the T-maze upon exiting the start arm, and were allowed to consume all pellets in both HD and LD arms of the maze before being returned to the start arm. Upon completion of this initial training, mice were only allowed to choose one arm of the maze; after the initial arm choice, the other arm was blocked. During 2 weeks mice choose between the two arms with no barrier in place. In the second training phase, a small barrier (6 cm high) was introduced in the HD arm for 1 week. Animals were then trained with the 12 cm barrier in the HD arm for the rest of the sessions. A training phase of 3 weeks was allowed before the drugs were administered. The test phase started at that moment: in experiments 5 and 6 animals received a single injection per week of caffeine (10 mg/kg) or saline vehicle, and of TBZ (4.0 mg/kg) or DMSO vehicle. These lower doses were chosen based on previous published data from our laboratory (27, 28) on the impact of caffeine and TBZ on running wheel activity, an activity that is highly reinforcing and requires a lot of vigor. Thus, TBZ 4.0 mg/kg and caffeine 10 mg/kg did not reduced running, but TBZ 8 mg/kg did (28). In addition, a pilot study demonstrated that caffeine (20.0 mg/kg) also reduced running wheel activity (*t*-test for independent samples [*t*_(9)_ = 6.83; *p* < 0.01], Saline = 1,982.3 ± 128.1, Caffeine 20.0 mg/kg = 1,309 ± 116.5). Based on these results, for experiments 5–7 we used lower doses of both drugs. In experiment 7 a different group of animals followed the same protocol and the pharmacological interactions between caffeine and TBZ were studied.

### Data analyses

All experiments but 2 and 4 used a within-groups design. Normally distributed and homogenous data form experiments 1 (A and B), 3 (A and B), and 7 were evaluated by repeated measures analysis of variance (ANOVA). Further analyses were conducted by non-orthogonal planned comparisons using the overall error term to assess differences between each dose and the control condition (29) (the number of comparisons was restricted to the number of treatments minus one). *T*-tests for dependent samples analysis were used for experiments 5 and 6. Experiments 2 and 4 used a between groups design and data were analyzed by *t*-tests for independent samples. Additionally, comparison of body weight progression was analyzed independently with repeated measures ANOVA for every group of animals in all these experiments, plus one-way ANOVAs were used to compare body weight between groups under different food access conditions (experiment 8). All data were expressed as mean ± SEM, and significance was set at *p* < 0.05. STATISTICA 7 software was used.

## Results

### Experiments 1 A and B. effect of caffeine on highly palatable food intake under habitual conditions: continuous or intermittent access

**Figure 2:**
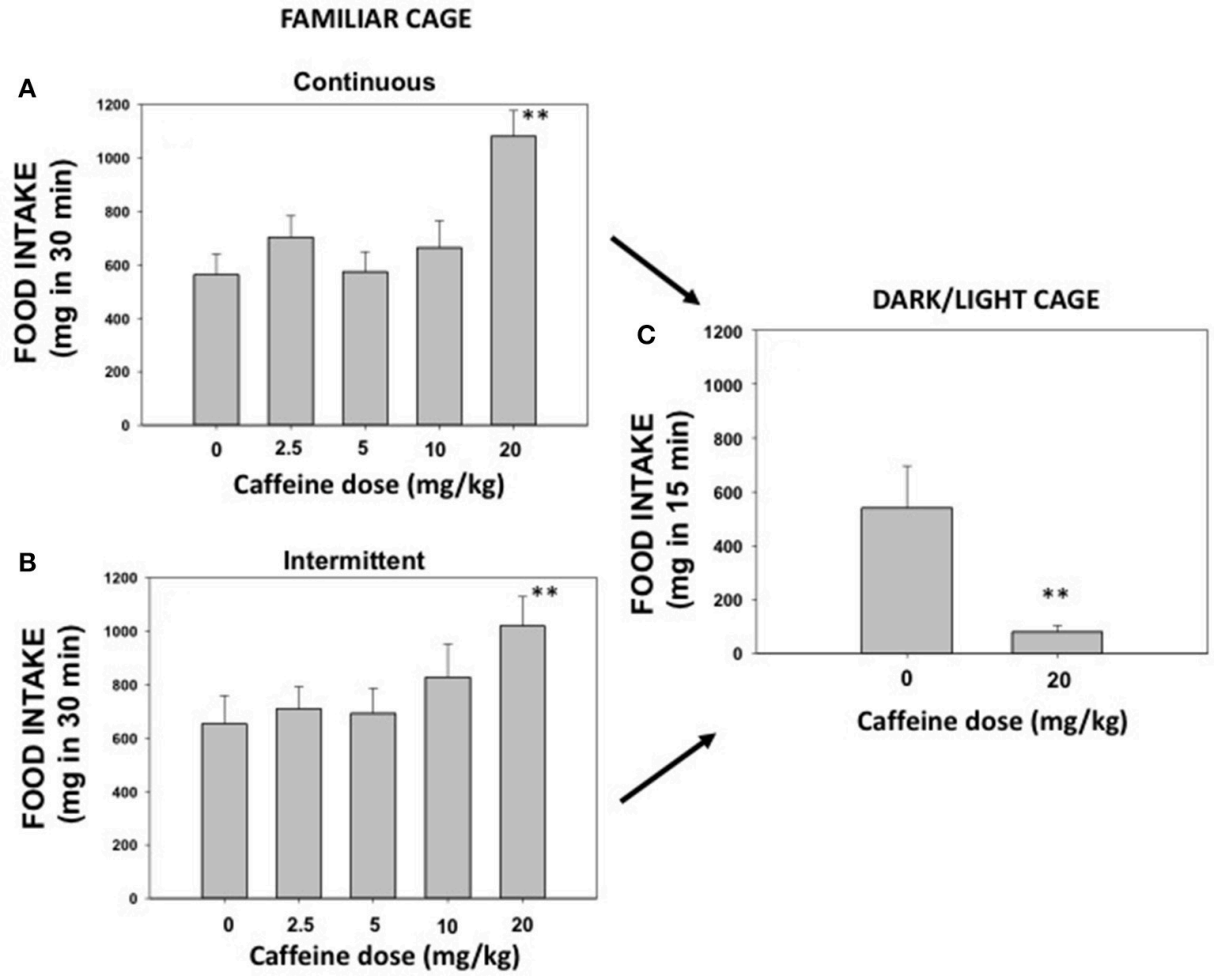
Effect of saline or caffeine (2.5, 5.0, 10.0, and 20.0 mg/kg) on palatable food intake in a familiar context under continuous access **(A)** or under intermittent access **(B)**, and effect of saline or caffeine (20.0 mg/kg) on food intake evaluated on a DL box **(C)**. Mean ± S.E.M. milligrams consumed. **p < 0.01 significantly different from vehicle.

The effect of caffeine (0, 2.5, 5.0, 10.0, and 20.0 mg/kg) on pellet intake was recorded during 30 min sessions. Repeated measures ANOVA for continuous access (*N* = 8) revealed a significant effect of caffeine [*F*_(4, 28)_ = 5.91; *p* < 0.01]. Planned comparisons showed that the highest dose of caffeine (20.0 mg/kg) significantly increased the amount of palatable food consumed (*p* < 0.01; Figure 2A). The repeated measures ANOVA for the intermittent access group (*N* = 8) revealed also a significant effect [*F*_(4, 28)_ = 4.77; *p* < 0.05], and the planned comparisons also confirmed that the highest dose of caffeine increased the amount of pellets consumed (*p* < 0.01; Figure 2B).

### Experiment 2. effect of caffeine on highly palatable food intake under anxiogenic conditions

The same mice used in the previous experiments were used in order to evaluate the effect of caffeine on pellet consumption under anxiogenic conditions. Because continuous and intermittent access exposition did not differ in the DL paradigm data from both groups were pulled to increase the number of animals (*N* = 16). Mice were split into two groups; one group received the dose of caffeine that had increased intake (20.0 mg/kg) in experiment 1 and the other group received saline. The *t*-test for independent samples revealed that this dose of caffeine significantly decreased food intake (mg in 15 min) in the DL paradigm [*t*_(16)_ = 2.90, *p* < 0.01; Figure 2C]. However, this dose of caffeine was not high enough to induce anxiety assessed with classical measures, such as time spent in the lit compartment [*t*_(16)_ = 0.098; n.s]. The saline group spent 118.4 ± 14.3, and the caffeine group spent 120.0 ± 8.6 s.

### Experiments 3 A and B. effect of TBZ on highly palatable food intake under habitual conditions: continuous or intermittent access

**Figure 3:**
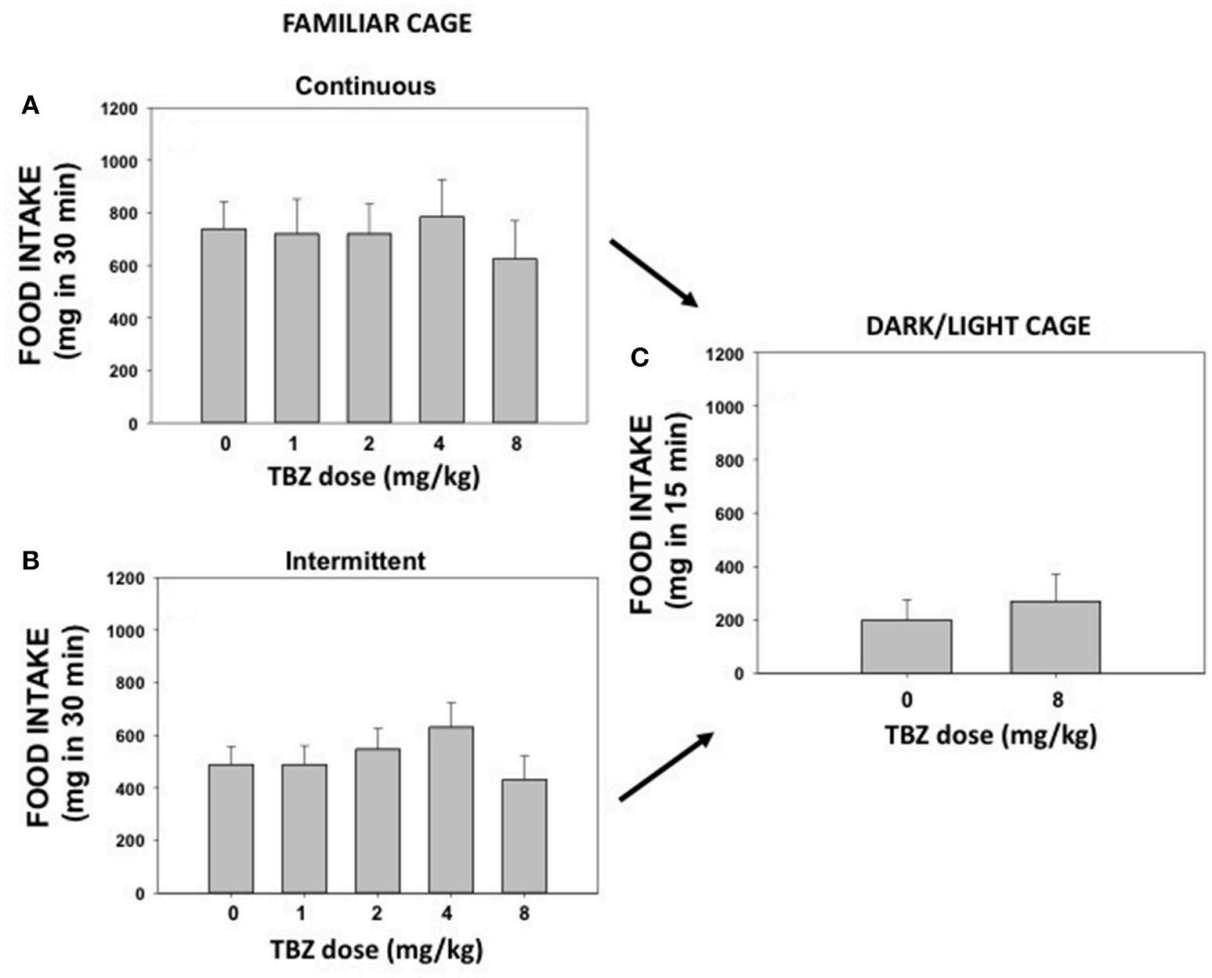
Effect of DMSO or TBZ (1, 2, 4, and 8 mg/kg) on palatable food intake in a familiar context under continuous access **(A)** or under intermittent access **(B)**, and effect of DMSO or TBZ (8 mg/kg) on food intake evaluated on a DL box **(C)**. Mean ± S.E.M. milligrams consumed.

The effect of TBZ (0, 1.0, 2.0, 4.0, and 8.0 mg/kg) on pellet intake was evaluated during 30 min sessions. Repeated measures ANOVA for continuous access (*N* = 7) did not show a significant effect of TBZ [*F*_(4, 24)_ = 0.51; n.s.] on amount of food consumed (Figure 3A). The repeated measures ANOVA for the intermittent access group (*N* = 7) revealed no significant effect either [*F*_(4, 24)_ = 1.34; n.s.; Figure 3B].

**Figure 4:**
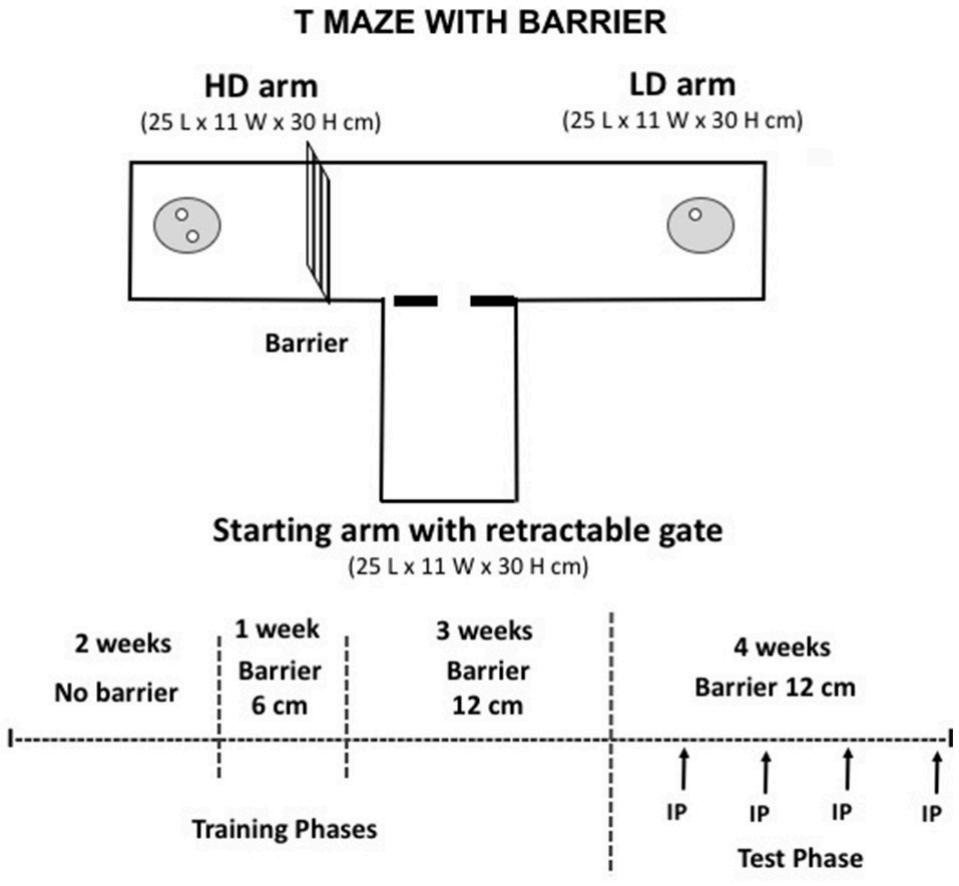
Schematic representation of the T-maze apparatus used in the present studies, and experimental procedure. All the surfaces and the doorway were constructed out of Plexiglas, and the barrier (depicted in the high-density arm, to the left) was constructed of wire mesh. The high density (HD) arm contained two food pellets, and the low density (LD) arm contained one food pellet.

### Experiment 4. effect of TBZ on highly palatable food intake under anxiogenic conditions

Mice used in experiments 3 A and B were used to evaluate the effect of TBZ on food intake in the DL box (Figure 3C). These animals (*N* = 14) from the continuous and intermittent groups were equally distributed into two treatment groups: DMSO vehicle or TBZ (8.0 mg/kg). The *t*-test for independent samples revealed no significant differences on total food intake between these two groups [*t*_(12)_ = 0.55; n.s.]. Classical measures of anxiety, such as time in the lit compartment, showed no significant effect of TBZ either [*t*_(12)_ = 0.54; n.s.]. The DMSO group spent 171.3 ± 16.8 s in the lit compartment, and TBZ group spent 184.4 ± 17.5 s.

### Experiments 5 and 6. effect of caffeine or TBZ on seeking of highly palatable food under effortful conditions

**Figure 5:**
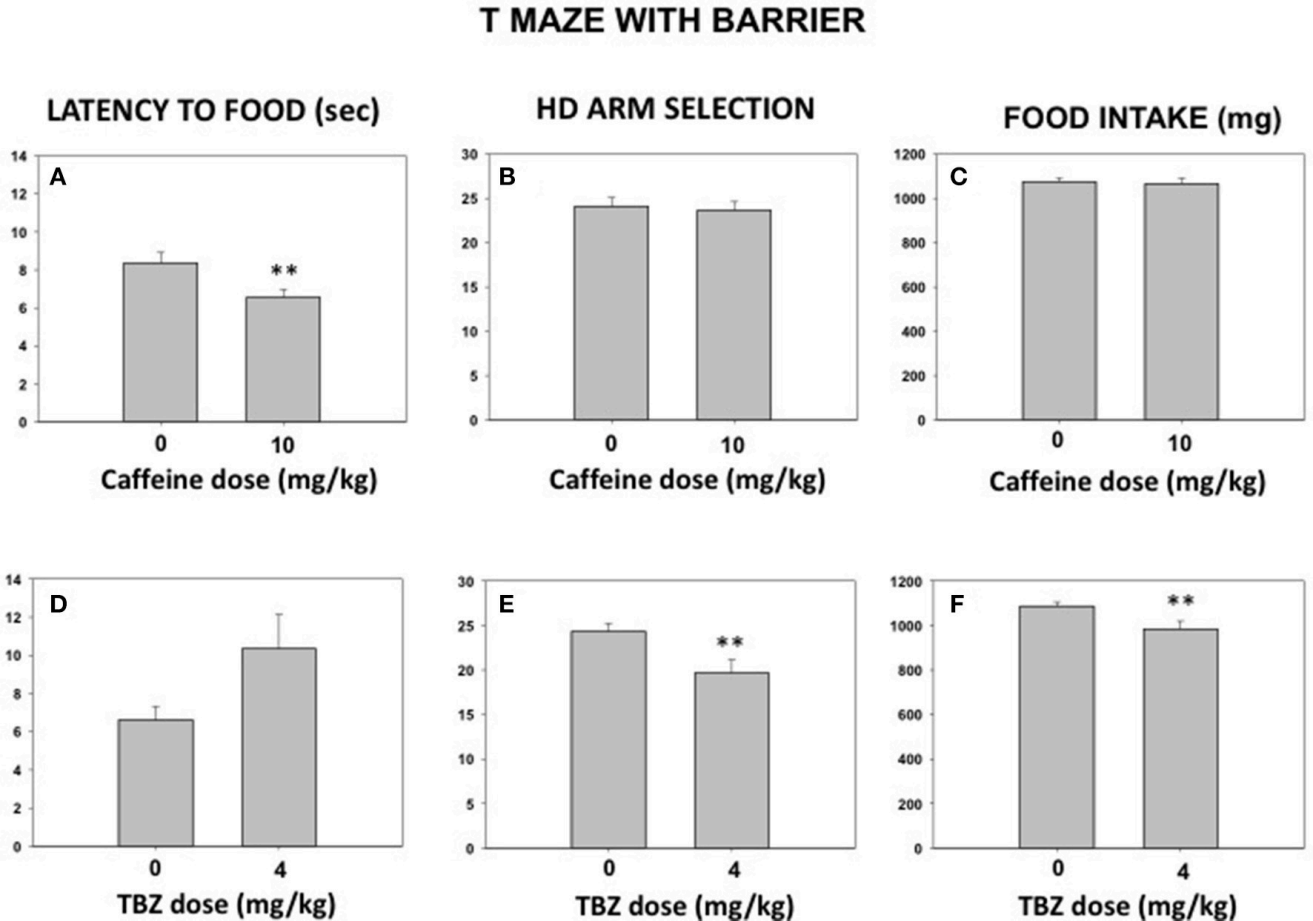
Impact of caffeine (vehicle or 10 mg/kg; **A–C**) or TBZ (vehicle or 4 mg/kg; **D–F**) on latency to food across 30 trials **(A,D)**, HD arm selection **(B,E)**, and total food consumed **(C,F)** in the T-maze with barrier. Mean (±S.E.M.) seconds from gate to food, number of arm choices in 30 trials and mg of food consumed. **p < 0.01 significantly different from their corresponding vehicle.

A new group of mice (*N* = 9) was trained in the T-maze. During the testing phase, some animals received caffeine (10.0 mg/kg) or its vehicle in the 2 first weeks and then TBZ (4.0 mg/kg) or its vehicle in the last 2 weeks and another group of animals received drugs in the reversed order. In addition, vehicle or drug treatments were administered in a randomized order. This experiment was also a pilot for the following study. The *t*-test for dependent samples showed that caffeine did not modify amount of food consumed [*t*_(8)_ = 0.38; n.s.] or selection of effortful options (HD arm) [*t*_(8)_ = 0.43; n.s.] to get it, although it reduced latency to reach the food across all the trials [*t*_(6)_ = 3.21; *p* < 0.01]. In contrast, 4.0 mg/kg TBZ did significantly reduced quantity of food consumed [*t*_(8)_ = 3.44; *p* < 0.01] because it reduced the number of HD arm choices [*t*_(8)_ = 3.92; *p* < 0.01]. However, there was no significant effect on latency to reach the food across trials [*t*_(7)_ = −1.71; n.s.]. Data can be seen in Figures 5A–F.

### Experiment 7. caffeine reversal of TBZ effects on seeking of highly palatable food under effortful conditions

**Figure 6:**
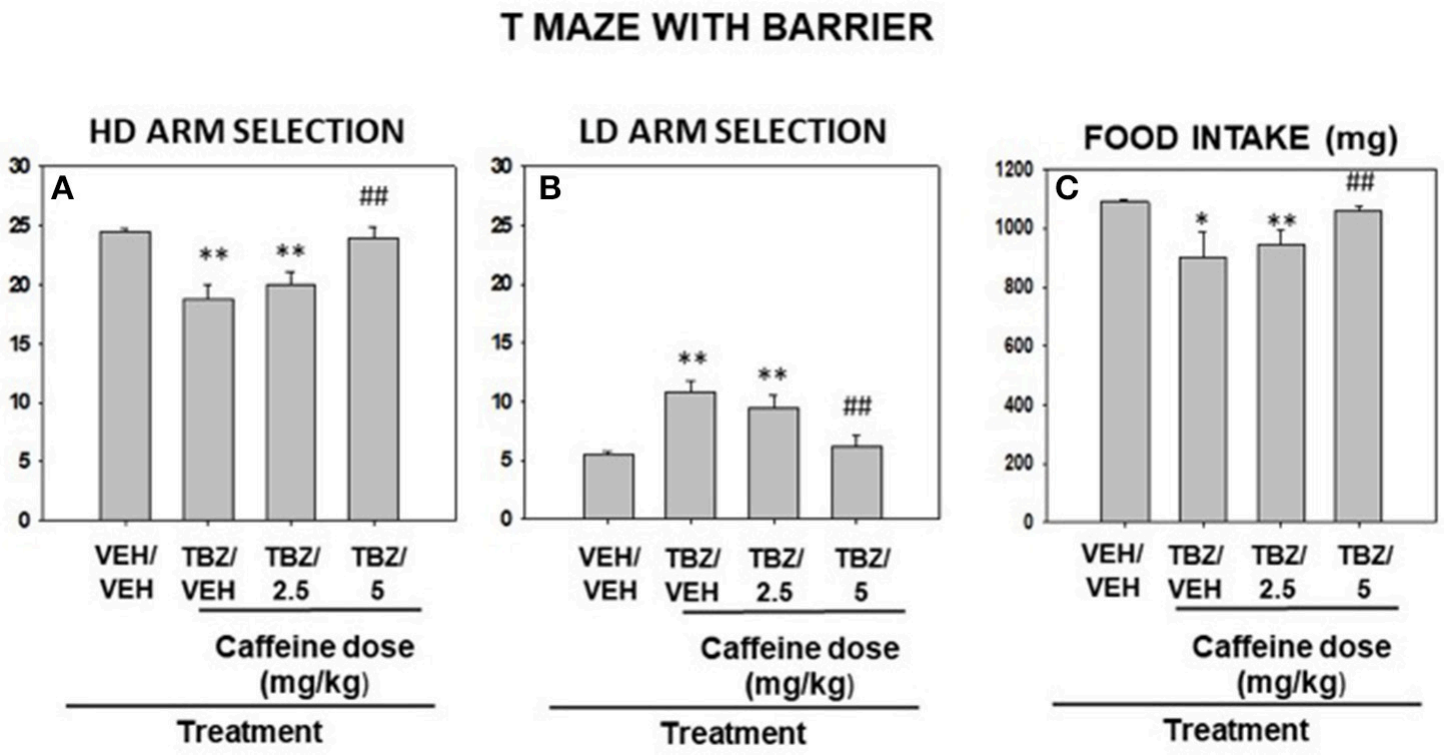
Effects of saline or caffeine (2.5 and 5.0 mg/kg) in mice co-administered with DMSO or TBZ (4 mg/kg) on HD **(A)**, LD **(B)** arm selection, and total food consumed **(C)** in the T-maze with barrier. Mean (±S.E.M.) number of arm choices in 30 trials and mg of pellets consumed. *p < 0.05; **p < 0.01 significantly different from VEH/VEH; ^##^*p* < 0.01 significantly different from TBZ/VEH.

Naïve mice (*N* = 8) received DMSO or TBZ (4.0 mg/kg) 120 min before test and saline or caffeine (2.5 or 5.0 mg/kg) 30 min before test (Figures 6A–C). Repeated measures ANOVA yielded an overall effect of drug treatment [*F*_(3, 21)_ = 11.91, *p* < 0.01] on HD arm selection. Planned comparisons showed that TBZ/VEH and TBZ/Caffeine 2.5 mg/kg condition were significantly different from VEH/VEH control condition (*p* < 0.01). In addition, co-administration of TBZ with the highest dose of caffeine (5.0 mg/kg) significantly increased HD arm selection (*p* < 0.01) compared to the TBZ/VEH condition, indicating an attenuation of the DA-depleting agent effects. LD arm selection was also modified in the reversal study. Repeated measures ANOVA indicated a significant effect of drug treatment [*F*_(3, 21)_ = 8.44, *p* < 0.01]. Planned comparisons showed that the TBZ/VEH and TBZ/Caffeine 2.5 mg/kg condition were significantly different from VEH/VEH control condition (*p* < 0.01), and TBZ plus caffeine (5.0 mg/kg) significantly decreased LD arm selection compared to TBZ/VEH condition (*p* < 0.01). The same pattern of results was found for the dependent variable mg of pellets consumed during the T-maze performance: a significant effect of drug treatment [*F*_(3, 21)_ = 4.15, *p* < 0.01], and a significant difference between VEH/VEH and TBZ/VEH (*p* < 0.01) on the one hand, as well as with TBZ/Caffeine 2.5 mg/kg on the other (*p* < 0.05). Moreover, TBZ/Caffeine (5.0 mg/kg) significantly restored mg consumed compared to TBZ/VEH condition (*p* < 0.01).

### Experiment 8. body weight progression under different food access conditions

**Figure 7:**
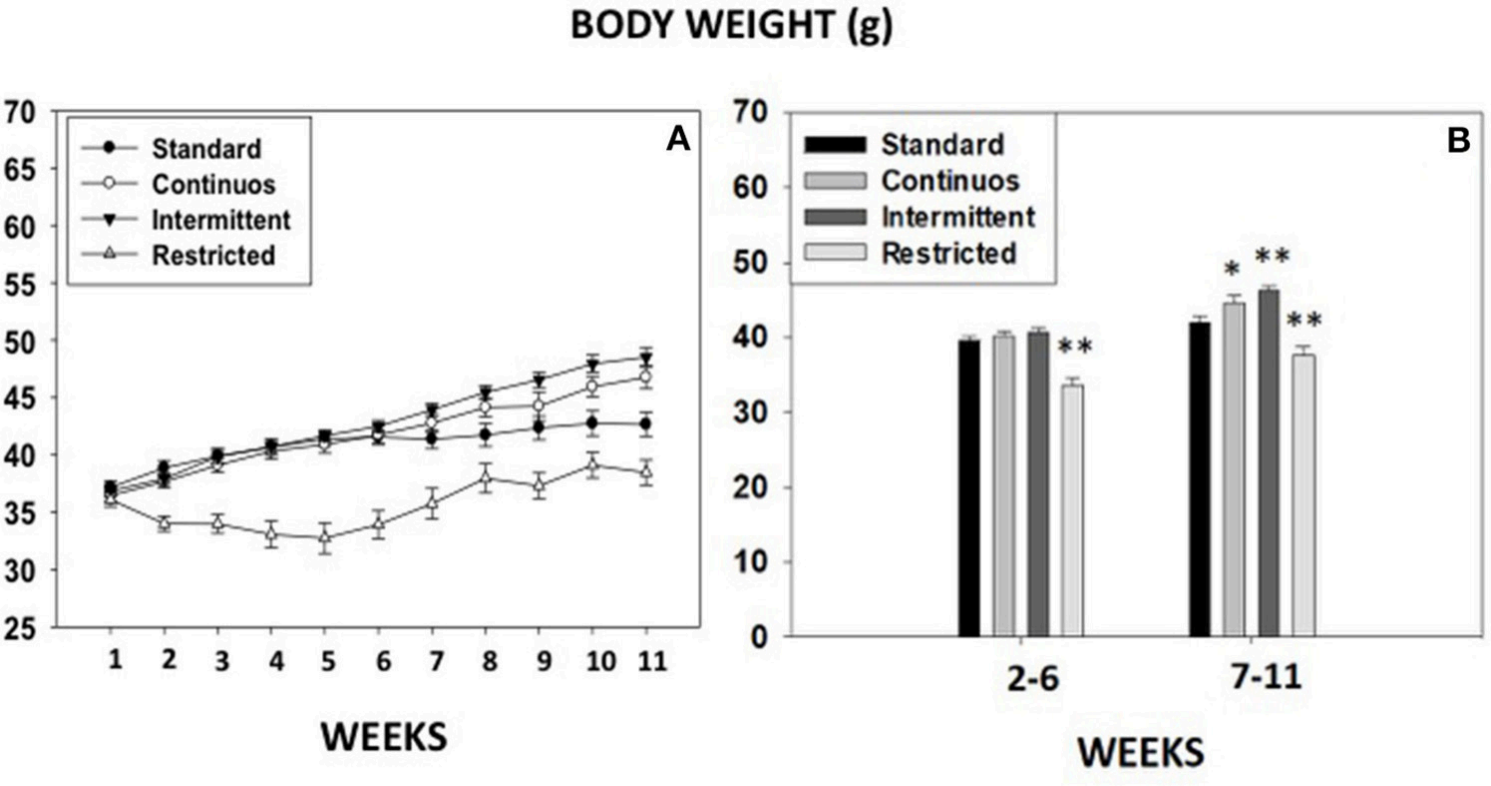
Body weight progression across different groups of mice exposed to different food access conditions. **(A)** Body weight (gr) gain from week 1 till week 11, and **(B)** average body weight of the four groups during two periods of 5 weeks each. Mean (±S.E.M.) weight in grams. *p < 0.05; **p < 0.01 significantly different from standard group.

Body weight progression from week 1 till week 11 from animals in experiments 1A-B, and 7 as well as a control group under standard house food conditions are shown in Figure 7. Independent repeated measures ANOVAs for body weight data, showed that the four groups of animals had a statistically significant progression in their body weight gain: standard control [*F*_(10, 140)_ = 18.15, *p* < 0.01], continuous access [*F*_(10, 140)_ = 91.55, *p* < 0.01], intermittent access [*F*_(10, 140)_ = 167.81, *p* < 0.01] and restricted access in the T-maze experiment [*F*_(10, 80)_ = 22.52, *p* < 0.01]. Planned comparisons indicate that animals in the standard group, in the continuous group (Experiments 1A + 3A), and in the intermittent group (Experiments 1B + 3B) increased body weight every week (*p* < 0.01). However, because of the home food restriction regime, animals in the restricted access condition (experiment 7), reduced significantly their initial body weight (< 15%) during the 5 first weeks, but by week 7, in spite of the food restriction, they had returned to their initial body weight, and progressively increased every week (*p* < 0.01) after that. Further comparisons using a between groups ANOVA for the average weight during weeks 2–6, or weeks 7–11, demonstrated that from week 2–6 there was a significant effect of type of food access on body weight [*F*_(3, 50)_ = 20.9, *p* < 0.01], and the planned comparisons showed that only the food restricted group in the T-maze experiment was significantly different from the standard group (*p* < 0.01). The one-way ANOVA for the average weight in weeks 7–11 also showed a significant effect [*F*_(3, 50)_ = 18.0, *p* < 0.01], but in this case, all groups were statistically different from the standard group (*p* < 0.05 for the continuous group, and *p* < 0.01 for the other two groups).

## Discussion

The present experiments indicate that the non-selective adenosine antagonist caffeine produces a complex pattern of effects on sweet food consumption depending on the conditions in which the food is presented to mice. In experiment 1 and 3 we have used two procedures of food access, in which after several weeks of daily or intermittent access to palatable food that is different from the standard chow, mice show high levels of consumption. There were no differences in food consumed or in body weight gain between the two access conditions. Considering that these animals are non-food restricted, an intake of around 600 mg of food in half an hour is remarkable. These results suggest that our procedures could have induced “binge eating,” which is characterized by excessive food intake during a short period of time, and typically is induced by offering a highly palatable food or fluid on a limited, or intermittent schedule (30–32). In addition, the higher dose of caffeine used (20.0 mg/kg) increased consumption of this palatable food to around 1,100 milligrams (25 pellets, 45 mg each) under both access conditions. The effect of caffeine is in agreement with a recent study in which acute caffeine (up to 26 mg/kg) increased standard chow consumption under habitual home conditions on the first 2 h (10). In that study, animals did not reach such a high level of intake (the maximum consumption of chow was 400 mg in 2 h). Thus, caffeine under repeated but limited access conditions, did not have an anorectic effect, but instead it potentiated patterns of binge eating for palatable food.

Because caffeine can have anxiogenic effects as well (27, 33), and anxiety and stress can affect food consumption, we explored the impact of the highest dose of caffeine (20.0 mg/kg) that was effective in increasing food intake, to evaluate its effects under anxiogenic conditions such as the bright open area of the classical DL box. Comparing animals that had received saline with those that had received the 20.0 mg/kg dose of caffeine, we observed an effect that was opposite to that seen in the previous experiment; caffeine decreased the amount of food consumed in 15 min. However, this dose of caffeine did not modify classical anxiety measures. This lack of anxiety effects is in accordance with previous experiments in mice using similar doses (10, 27). Thus, although caffeine at this dose did not show signs of being anxiogenic in terms of exploration in the DL box, it did reduce food consumption, showing that this variable is possibly more sensitive to the anxiogenic properties of caffeine.

The patterns of results observed after caffeine administration were not observed after a broad range of TBZ doses that had previously been demonstrated to reduce DA in ventral striatum of mice (28) and, more specifically, in Nacb core of rats (34). TBZ depletes DA in the central nervous system by reversibly inhibiting the vesicle monoamine transporter type 2 and preventing monoamine uptake into presynaptic neurons (35). Thus, in the present results, DA depletion did not affect consumption of food with a high content of sucrose, neither in a familiar context nor in an anxiogenic one when there is no work or effort involved in the access to the food.

As a psychostimulant, caffeine can also affect locomotion and exploration. Increases in locomotion with moderate doses of caffeine are seen in studies were a low baseline activity is seen, such as mice habituated to an open field (27, 33). However, in situations of high baseline levels such as voluntary running, high levels of locomotion are easier to decrease (27). Thus, for the following T-maze effort conditions in which exploration, voluntary running and barrier climbing are required, lower doses of caffeine were used. It has been demonstrated that the T-maze is a good tool to measure effort-related decision-making in rodents (26, 36, 37), and that this paradigm is sensitive to behavioral manipulations such as pre-feeding, which devalues food reinforcement by reducing appetite and food motivation increasing omissions (26). As seen in the present results (Figures 5A–C) caffeine (10.0 mg/kg) decreased latency to reach the food independently of the presence or absence of the barrier. However, this dose did not affect preference for the arm that contained more pellets and did not increase omissions (data not shown), which resulted in no changes in pellets earned and consumed. The pattern of results was quite different when anergia was induced by administering the DA-depleting agent TBZ. At this dose, TBZ did not have a significant effect on latency to reach the food, although some animals did show increased latency. TBZ-treated animals reduced selection of the HD arm but compensated by increasing the selection of the LD arm (see Figure 6B). This treatment did not change appetite since animals did not increase the number of omissions and eat all the pellets that they earned, although they were significantly fewer (see Figures 6F, 7C). Interestingly, co-administration of a relative low dose of caffeine (5.0 mg/kg), reversed this anergia inducing effect of TBZ. This reversal of TBZ effects by caffeine is probably due to adenosine-DA receptor interaction having opposite effects on the adenylyl cyclase-related signal transduction cascade. Thus, caffeine acting on A_1_ receptors would reduce the impact of D_1_ receptors located in the substance *P* containing neurons of Nacb, and the same would be true for caffeine acting on the A_2A_ receptor co-localized with D_2_ receptors in enkephaline containing neurons (21–23).

In operant paradigms that require low effort (variable or fixed intervals schedules) high doses of caffeine (≥ 50.0 mg/kg), reduce lever pressing for chow pellets (11), but doses up to 20.0 mg/kg increased lever pressing although there was no net increase in access to highly palatable food (12). In operant schedules that require high effort (fixed ratio 20 or progressive ratio schedules), doses up to 40.0 mg/kg of caffeine reduced lever pressing for palatable food (12), and in contrast, caffeine (up to 25.0 mg/kg) improves performance in animals responding for sucrose solutions (13, 14, 38).

In summary, caffeine administered acutely to mice at moderate to high doses (27) can potentiate binge eating when subjects have already stablished a pattern of excessive eating. However, this same dose led to clear reductions in food consumption if the context is prone to increase anxiety levels (i.e., the DL box). Although lower doses of caffeine do not change appetite and do not impair orientation toward food under effortful conditions, they help to approach the food by improving speed and by reversing performance to normal levels when anergia/ psychomotor retardation was induced by DA-depletion.

This pattern of results was quite different for TBZ. Although TBZ shifted choice behavior in the T-maze, the food consumption studies showed that TBZ had no effect on food intake. Thus, TBZ-induced shifts in effort-related choice do not appear to be due to changes in primary food motivation, the unconditioned reinforcing properties of food, or food preference. These data support previous results indicating that Nacb DA depletions and TBZ do not substantially impair appetite for food, or produce a general disruption of all aspects of primary food motivation (34, 39–41). Nacb DA depletions did not reduce food intake or feeding rate, nor did they impair food handling (40). However, depletion of mesolimbic DA has been reported to produce impairments in behavioral activation, and, more specifically, in effort-related aspects of food motivation (16). The shift in choice induced by DA depletion or antagonism in animals tested in the T-maze barrier task has been demonstrated to be a valid rodent model of psychomotor slowing, fatigue and anergia (24, 26, 36, 37, 42–45). After DA depletions animals shift their behavior toward the lower effort options, however, they are still oriented toward the food. Moreover, previous work has also demonstrated that, if animals do not have an option because both arms have a barrier or because there is no food in the no-barrier arm, animals perform at control levels, demonstrating that DA depletion or antagonism does not block the absolute motor capacity of the animal to climb the barrier, and does not reduce general food motivation (24, 26, 37, 42).

A DA system that also has been implicated in food motivation is the innervation of the lateral hypothalamus (LH). LH DA sources include local hypothalamic monoaminergic neurons expressing enzymes for DA synthesis (46), as well as ascending projections from the Ventral Tegmental Area (VTA) (47). It has been suggested that DA release in the LH regulates the consummatory component of feeding behavior (48). Thus, DA increases during feeding in proportion to meal size (49), and to the appearance of satiation processes (48, 50). Furthermore, low sucrose intake was associated with increased VTA DA (51), and DA administration into the LH produced anorectic effects (52, 53). However, although we cannot completely discount the idea that TBZ could be having an impact on LH neurons, the lack of results on food consumption after TBZ administration in experiments 3A-C, when mice had direct access to the food, and the clear impact of TBZ in experiments 6 and 7 when an effortful approach is essential to get access to food, indicates that our experimental conditions are more related to Nacb DA than LH DA. Moreover, because the hypothalamus in rodents seems sparse in adenosine receptors (54, 55), it does not seem likely that caffeine could be acting on LH neurons to modulate palatable food intake.

The present work has potential clinical relevance, since appetite is impaired in many disorders such as anorexia and bulimia, anxiety and depression. In addition, effort-related motivational symptoms such as anergia, fatigue, and psychomotor slowing seen in depressed humans are very resistant to classical antidepressant treatments such as 5-HT uptake inhibitors (56, 57), and caffeine has been demonstrated to enhance the antidepressant-like activity of common antidepressant drugs (58). Moreover, caffeine is an antagonist at both A_1_ and A_2A_ adenosine receptors, and evidence indicates that selective A_2A_ receptor antagonists may be useful as treatments for motivational deficits seen in depression and other disorders (16, 36). Further research on the motivational effects of adenosine antagonists may contribute to a greater understanding of the neural mechanisms mediating various aspects of motivation.

## Author contributions

MC and JS designed, supervised the experiments and wrote the manuscript. NS, LL-C, CC-R, and RO-G performed the experiments, contributed to the analysis of data and the writing of the methodology and the results.

### Conflict of interest statement

The authors declare that the research was conducted in the absence of any commercial or financial relationships that could be construed as a potential conflict of interest.
